# EndNote for iPad (version 2.4)

**DOI:** 10.5195/jmla.2017.191

**Published:** 2017-07-01

**Authors:** Michelle Demetres

## OVERVIEW

EndNote for iPad is the tablet companion to the EndNote citation manager from Clarivate Analytics (formerly produced by Thomson Reuters). Similar to its sister products, EndNote for iPad allows users to manage, store, annotate, and share their collected references. The app is designed to easily sync users’ libraries across platforms so that users can utilize the mobility of the iPad while still being able to work from the same reference set.

## FEATURES

Upon opening the app, users are prompted to log into an EndNote account or create a new, free account via myendnoteweb.com. Once logged in, users can sync accounts, search databases, import new citations, organize citations into groups, attach full text, and annotate portable document format (PDFs) files.

## CITATION MANAGEMENT

Users can pull citations directly from the web via EndNote for iPad’s builtin web browser. This browser can be used to access any website but has direct links to several databases, including Web of Science, PubMed, and Google Scholar. “Direct export” functions that are commonly found in databases, such as Export to EndNote or Save to Bibliography Software, are supported in the app [1]. Most .ciw, .ris, .enw, and .nbib files can also be imported into the EndNote for iPad library. These citations can then be edited, including the ability to type comments into the notes field. They can also be shared via email, with or without attachments.

From the app’s main library page, users can manually sync accounts, or accounts can be synced automatically at regular intervals so that all platforms mirror each other. References, attachments, and PDF annotations are synced across all platforms: EndNote Web as well as Mac and Windows desktop clients. The last sync time and date is displayed in the upper-left corner of the main library view of the app [1].

EndNote for iPad does not have direct “Cite While You Write” functionality; however, there are a few workarounds in place that can aid in manuscript writing. By selecting “Copy citation,” a full, formatted, final bibliographic citation will be copied to the clipboard. For in-text citations, selecting “Copy temporary citation” creates an end-of-sentence citation in the format of {First Author Last Name, Year} and copies it to the clipboard. This temporary citation can be pasted into a manuscript and later formatted using EndNote Cite While You Write for Windows, Mac, or online [1].

Citations can also be organized into groups, just as with the desktop and online versions. Any existing reference groups will be carried over after a sync is initiated. Group tags can be dragged to reorder them, and any reference can be in any number of groups.

The EndNote for iPad app also offers linked keywords, a feature not found in the desktop client or EndNote Web. Each keyword attached to a reference is clickable and shows all other references in the library that have been indexed with the same keyword.

## WORKING WITH PORTABLE DOCUMENT FORMAT FILES (PDFS)

PDF full text can be downloaded and attached to references without leaving the app. Whether downloaded via the browser or opened from an email attachment, files are saved to the “Downloads” tab in the main library view. These files can then be attached to individual references. PDF files can also be imported from external storage accounts, including Dropbox, Google Drive, and OneDrive.

Attachments are not limited to PDF files. Most file types supported by iOS can be used in EndNote for iPad, including graphics and video files. Users can swipe horizontally to move between multiple files attached to the same reference. PDF files can also be opened in other iPad applications that support PDF files. Attachments can be downloaded for offline availability to allow reading, annotating, and editing without an Internet connection. All references in the “Favorites” group will automatically be downloaded for offline availability.

Perhaps the most robust tool in the EndNote for iPad app is its expanded functionality for annotating PDF files. Shape, drawing, and text tools are available to annotate PDFs through the integrated toolbar. These tools are customizable, including the size, color, opacity, font faces, and alignment [1]. Annotations are automatically saved and can be removed at any time by clicking on the annotation and deleting it. These annotated PDF files can then be printed, shared with colleagues, and synced across all other EndNote platforms, which allows users to create and edit the same PDF annotations from anywhere.

## TECHNICAL REQUIREMENTS

The EndNote app is currently only available for iPad and is not compatible with other tablet devices. It is available for free download in the iTunes App Store and requires iOS 9.0 or later. Users do not need an existing EndNote account to use the app, although they will be prompted to register for a free online account upon opening the app after downloading it [2]. The functionality in the iOS app is the same for users of free accounts as it is for users who have purchased the desktop application.

## USABILITY

Syncing EndNote for iPad with the desktop client and online version is easy and mimics the process across platforms. The web interface for collecting new citations is seamless and intuitive, and the workflow is familiar to those who have previously used EndNote or other citation managers. Reading and annotating PDFs is similarly easy, with the standard scrolling interface for reading and the ability to hide the annotating toolbar.

Less intuitive is attaching full text to citations. In the desktop client, EndNote has the ability to find full text and automatically attach it to the correct citation. In EndNote for iPad, unless the PDF is already attached to the record via platform sync, full text must be separately downloaded and individually attached to each record. This process is clumsy and time consuming when users are working with a large number of citations; I recommend attaching full text via the desktop client and then syncing accounts.

EndNote for iPad’s lack of direct support for Cite While You Write is a significant drawback. While there is a workaround for this process, it adds unnecessary steps that make it an unrealistic option for manuscript writing.

## SUPPORT

EndNote for iPad’s support documentation is robust and easily accessible. Users can click on the settings button while in the main library view to access the Getting Started Guide, which opens as a PDF in the attachment viewer. A “Contact Us” link takes usres to EndNote’s email form on the web, where users can access technical support, a product suggestion forum, and how-to videos on EndNote’s You-Tube channel.

## SUMMARY

EndNote for iPad is most useful for collecting citations and annotating PDFs on-the-go, providing an excellent way to read and annotate literature while keeping it organized and centralized. EndNote for iPad is a useful companion tool to the existing End-Note products, but it is by no means a stand-alone replacement for the desktop client or online version.
